# Clinical efficacy and safety of neoadjuvant chemotherapy with paclitaxel and cisplatin in combination with concurrent chemoradiotherapy for locally advanced cervical cancer: a systematic review and meta-analysis

**DOI:** 10.1093/jrr/rrae073

**Published:** 2024-10-05

**Authors:** Penpa Yeshe, Fang Li

**Affiliations:** Department of Obstetrics and Gynecology, Shanghai East Hospital, School of Medicine, Tongji University, No. 150, Jimo Road, Pudong New Area, Shanghai 200120, P.R. China; Department of Obstetrics and Gynecology, Shanghai East Hospital, School of Medicine, Tongji University, No. 150, Jimo Road, Pudong New Area, Shanghai 200120, P.R. China

**Keywords:** neoadjuvant chemotherapy, concurrent chemoradiotherapy, locally advanced cervical cancer, meta-analysis

## Abstract

The meta-analysis was to evaluate the therapeutic benefits of neoadjuvant chemotherapy (NACT), primarily consisting of platinum-based regimens in conjunction with paclitaxel, when integrated with concurrent chemoradiotherapy (CCRT) for individuals afflicted with locally advanced cervical cancer (LACC). The outcomes were determined by overall survival (OS), progression-free survival (PFS), complete response rate (CRR), objective response rate, recurrence rate and adverse events. The assessment of these outcomes was based on the relative risk (RR) accompanied by its 95% confidence interval (CI). Eight articles were included for analysis. LACC patients who underwent treatment with paclitaxel combined with cisplatin (TP)-based NACT in conjunction with CCRT demonstrated improved OS at 2 (RR: 1.11, 95% CI: 1.07, 1.16, *P* < 0.001), 3 (RR: 1.30, 95% CI: 1.23, 1.37, *P* < 0.001) and 5 years (RR: 1.20, 95% CI: 1.10, 1.32, *P* < 0.001), as well as PFS at 1 (RR: 1.03, 95% CI: 1.00, 1.06, *P =* 0.035), 2 (RR: 1.21, 95% CI: 1.04, 1.40, *P* = 0.012), 3 (RR: 1.26, 95% CI: 1.17, 1.34, *P* < 0.001) and 5 (RR: 1.39, 95% CI: 1.25, 1.55, *P* < 0.001) years, when compared with patients who received CCRT alone. Moreover, the TP-based NACT in conjunction with CCRT achieved a higher CRR and exhibited a lower rate of disease recurrence (RR:1.28, 95% CI:1.08, 1.50, *P* = 0.003). No significant differences in the risk of adverse effects including anemia, leukopenia, thrombocytopenia, radiocystitis and radiation enteritis between the group treated with TP-based NACT combined with CCRT and the group treated with CCRT alone were observed. The combination of TP-based NACT and CCRT demonstrates superior clinical efficacy than CCRT alone. This study may contribute to reducing the burden of LACC by using TP-based NACT plus CCRT.

## INTRODUCTION

Cervical cancer ranks as the fourth leading cancer among women globally, resulting in over 300 000 deaths worldwide [1]. Approximately 53.5% of cervical cancer cases are diagnosed at the locally advanced stage, with a 5-year relative survival rate for these stages ranging from 24.0 to 76.1% [2]. The established clinical standard for treating locally advanced cervical cancer (LACC) is concurrent chemoradiotherapy (CCRT) [3]. The 5-year overall survival (OS) for women with LACCs is 70% following completion of CCRT [4]. Despite the CCRT treatment, there remains a significant recurrence rate, with 40% of patients experiencing a relapse of the tumor [5]. Further refinement and exploration of novel therapeutic strategies may be warranted to improve the efficacy of treatment. 

In 1982, the concept of neoadjuvant chemotherapy (NACT) was initially introduced by Frei [6]. NACT has emerged as a promising frontier in managing cervical cancer [7]. Currently, the combination of NACT with CCRT is frequently utilized in clinical practice, but its application value remains controversial. A randomized controlled study suggests that, compared with CCRT alone, the use of paclitaxel combined with cisplatin (TP)-NACT followed by CCRT may improve treatment completion rates, enhance the rate of complete remission (CR) and the OS rates at 1 and 2 years, and reduce the incidence of distant metastases in patients with LACC with larger tumor burdens [8]. A phase III trial has found that administering dose-dense TP on a weekly basis, followed by standard CCRT, can achieve favorable response rates [5]. However, a randomized phase II trial has found that treating LACC, adding NACT consisting of cisplatin and gemcitabine to the standard CCRT regimen did not lead to better outcomes compared with CCRT alone and may even be linked to a worse prognosis [9]. For patients with unselected LACC, adjuvant chemotherapy with carboplatin and paclitaxel following standard cisplatin-based CCRT may increase short-term toxicity without improving OS [10]. In addition, platinum-based combination therapy with paclitaxel is currently the preferred regimen in the NACT setting for [5, 11]. Therefore, a meta-analysis of the clinical efficacy of NACT (platinum-based combination therapy with paclitaxel) combined with CCRT for the treatment of LACC is warranted.

Herein, the purpose of this meta-analysis was to assess the clinical efficacy of NACT, particularly platinum-based combination therapy with paclitaxel, when used in conjunction with CCRT for patients with LACC.

## MATERIALS AND METHODS

This study followed the guidelines set by PRISMA (Preferred Reporting Items for Systematic Reviews and Meta-Analyses) [12].

### Data sources and search strategy

An extensive literature search was performed across a range of databases, comprising PubMed, Embase, the Cochrane Library and Web of Science, in addition to the China National Knowledge Infrastructure, Wanfang and Weipu (VIP) databases. The search period extended from the earliest available records in these databases to 30 December 2023. The retrieved literature was imported into EndNote20 for initial screening. The initial phase of selection involved examining the titles and abstracts of the identified literature. After preliminary filtration, a thorough evaluation of the full-text articles was conducted to eliminate any that failed to align with the established inclusion criteria for the study. The remaining articles, after this process of exclusion, were then included in the current study. The detailed PubMed search strategy is shown in Supplementary File 1.

### Inclusion and exclusion criteria

Inclusion criteria were established according to the Population, Intervention, Comparator, Outcome, and Study design (PICOS) framework, which included considerations for: (i) P: population- studies involving patients diagnosed with LACC; (ii) I: intervention-TP-based NACT in combination of CCRT; (iii) C: comparator-CCRT; (iv) O: outcome- OS at 1, 2, 3 and 5 years, progression-free survival (PFS) at 1, 2, 3 and 5 years, complete response rate (CRR), objective response rate (ORR), recurrence rate and adverse events; (v) S: study design - cohort studies, and randomized controlled trials (RCTs); (vi) studies published in English and Chinese.

The following exclusion criteria have been established: (i) animal studies; (ii) retracted studies; (iii) reviews, meta-analyses, guidelines, errata, case reports, books, conference abstracts, editorial materials, letters and trial registration records; (iv) irrelevant topics.

### Data collection and assessment of quality of studies

Two independent investigators conducted the data extraction process meticulously, ensuring that a systematic collection of the following information was obtained from eachstudy: authors, study design, the year the study was published, country of enrolment, sample size, age, type of cancer, cancer staging, intervention frequency, follow-up duration and outcomes.

The quality assessment of the cohort studies was conducted using the Newcastle-Ottawa Scale (NOS) [13]. This scale evaluated the quality through three main components consisting of eight items in total, focusing on the selection of the study population, comparability between groups, and the exposure or outcome assessment. The scoring system of the NOS ranges from 0 to 9, with the quality of studies being classified into low (0–3 points), moderate (4–6 points) and high (7–9 points) quality tiers. For the evaluation of RCTs, the Cochrane Risk of Bias Assessment Tool [14] was employed. This tool thoroughly evaluated the potential for bias across six distinct areas: selection bias, performance bias, detection bias, attrition bias, reporting bias and other types of biases. For each of these criteria, a judgment is made using a standardized scale that classifies the risk of bias as ‘low’, ‘unclear’ or ‘high’, ensuring clarity and consistency in the assessment.

### Statistical analysis

All outcome data were subjected to statistical analysis using Stata 15.0 software. The effect size utilized in the analysis was the relative risk (RR), accompanied by its corresponding 95% confidence intervals (CIs). The I^2^ statistic was used to evaluate the consistency of findings among studies for each outcome measure. When substantial heterogeneity was identified, with an I^2^ value equal to or exceeding 50%, the analysis was conducted using a random-effects model. On the other hand, in the absence of significant heterogeneity (I^2^ < 50%), a fixed-effect model was employed. The threshold for statistical significance was defined as *P* < 0.05.

## RESULTS

### Study selection process and characteristics of included studies

**Fig. 1:**
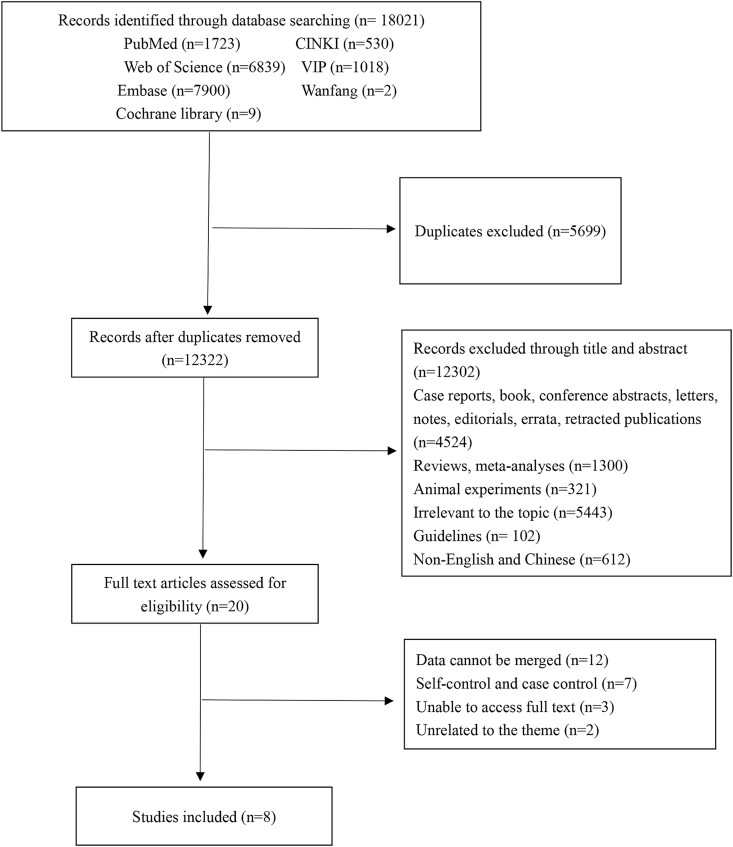
The diagram flow of study selection process.

A total of 18 021 records were identified from a range of databases. After identifying the records, duplicates were removed, leaving 12 322 records. By reviewing the titles and abstracts, 12 303 records remain. After the full-text assessment, 19 articles were identified. Ultimately, based on the evaluation, eight articles [8, 9, 15–20] fulfilled all the inclusion criteria and were consequently incorporated into the meta-analysis and systematic review. Figure 1 illustrates the process of the selection of relevant literature for the study. Of the included studies, seven are RCTs, and one is a cohort study. The studies were primarily conducted in China. Table 1 provides a summary of the characteristics of the studies that were included in the analysis. The one cohort study had a NOS score of 7, indicating a high quality (Supplementary File 2). The risk of bias graph of RCTs is shown in Supplementary Fig. 1 and the risk of bias summary of included RCTs is depicted in Supplementary Fig. 2.

**Table 1:** Characteristics of included studies

Author	Year	Study design	Country	Tumor type	Pathological type	Treatment	Therapy	Age(year)	Sample size (*n*)	Follow-up period (month)	Outcome
Li, F	2023	RCT	China	FIGO stages IB3 and above	SCC, AC, ASC	NACT (TP) + CCRT	NA	51 (35–69)	73	12	OS, PFS, ORR,CRR, metastasis rate, recurrence rate, death, anemia, leukocytopenia, thrombopenia, radiation enteritis, radiocystitis
						CCRT		53 (30–68)	68	12	
Da Costa	2019	RCT	Brazil	IIB-IVA	SCC, AC, UC	NACT (PG) + CCRT	Once a week	48 (22–69)	55	36	OS, PFS, ORR, CRR, recurrence rate, anemia, emesis, leukocytopenia, nausea, thrombopenia
						CCRT		45 (20–67)	52	36	
Li, N	2019	Cohort	China	FIGO stages IB3 and above	SCC	NACT (TP) + CCRT	NA	51.44 ± 8.75	85	38	OS, PFS
						CCRT		55.48 ± 11.65	46	38	
Zhong	2020	RCT	China	III-IVA	SCC, AC, ASC	NACT (IP) + CCRT	Once a week	49.3 ± 5.42	31	NA	ORR, CRR, anemia, leukocytopenia
						CCRT		49.26 ± 5.48	31	NA	
Tang, J	2012	RCT	China	IIB-IVA	AC, ASC	NACT (TP) + CCRT	Twice a week	53.6 (36–69)	440	60	OS PFS, ORR, CRR, recurrence rate, anemia, leukocytopenia, thrombopenia, radiocystitis
						CCRT		58.7 (35–68)	440	60	
Tripathi	2019	RCT	India	IIB-IIIB	SCC	NACT (TP) + CCRT	5 times a week	46.85 ± 8.45	40	6	ORR, CRR, anemia, emesis, leukocytopenia nausea
						CCRT		47.13 ± 10.28	40	6	
Tian	2017	RCT	China	IIA-IVA	SCC, AC, ASC	NACT (TP) + CCRT	NA	55.15 (28–69)	48	42	OS, PFS, radiation enteritis, radiocystitis
						CCRT		55.15 (28–69)	46	42	
Jiang	2014	RCT	China	II-III	SCC, AC, ASC	NACT (TP) + CCRT	NA	41.5 ± 2.8	28	36	ORR, CRR
						CCRT		43.6 ± 1.8	28	36	

Notes: RCT = randomized controlled trial, SCC = squamous cell carcinoma, AC = adenocarcinoma, ASC = adenosquamous carcinoma, UC = undifferentiated carcinoma, TP = cisplatin and paclitaxel, PG = cisplatin and gemcitabine, IP = cisplatin and irinotecan, NACT = neoadjuvant chemotherapy, CCRT = concurrent chemoradiotherapy, OS = overall survival, PFS = progression-free survival, ORR = objective response rate, CRR = complete response rate.

### Meta-analysis of the clinical efficacy and safety of TP-based NACT combined with CCRT in patients with LACC

#### The comparison of OS between LACC patients treated with TP-based NACT combined with CCRT and CCRT

**Fig. 2:**
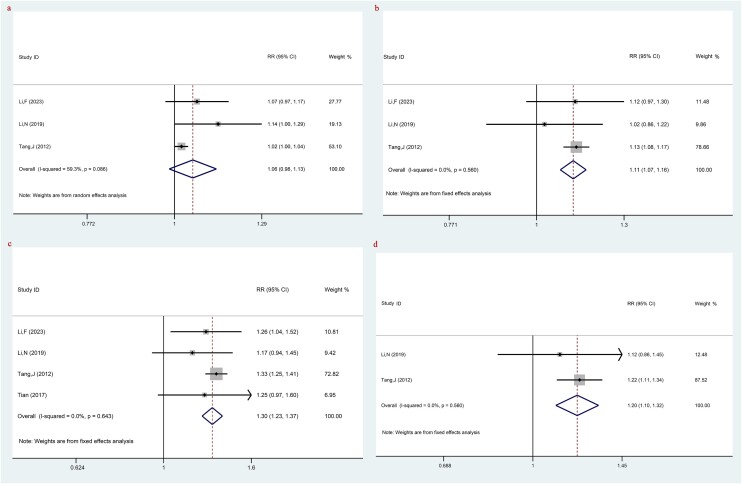
The comparison of OS between LACC patients treating with TP-based NACT combined with CCRT and CCRT; (**a**) 1-year OS; (**b**) 2-year OS; (**c**) 3-year OS; (**d**) 5-year OS. Each row represents an individual study, with labels indicating the study name and year. The bottom row labeled ‘Overall’ represents the combined results of all studies. The horizontal lines for each study represent the 95% CI, and the square symbols indicate the RR. The size of the square is proportional to the study’s weight, with larger squares indicating greater weight. The I^2^ statistic is included to show the percentage of variability across studies due to heterogeneity. The dashed line is placed at the position of 1, representing the null hypothesis of no effect (i.e. no difference between the treatment and control groups). If a study’s CI crosses this line, it suggests that the study’s result is not statistically significant.

The comparison of 1-year OS between LACC patients treated with TP-based NACT combined with CCRT and CCRT was assessed by three studies. After heterogeneity testing with an I^2^ of 59.3%, a random-effects model was employed for analysis. The results indicated that there was no significant difference between TP-based NACT combined with CCRT and CCRT in 1-year OS (RR: 1.06, 95% CI: 0.98, 1.13, *P* = 0.127) (Fig. 2a, Table 2).

**Table 2:** Meta-analysis of the clinical efficacy and safety of TP-based NACT combined with CCRT in patients with LACC

Outcome	Index	N of studies	RR (95% CI)	*P*	I^2^
One year OS	Overall	3	1.06 (0.98, 1.13)	0.127	59.3%
Two years OS	Overall	3	1.11 (1.07, 1.16)	<0.001	0.0%
Three years OS	Overall	3	1.30 (1.23, 1.37)	<0.001	0.0%
Five years OS	Overall	3	1.20 (1.10, 1.32)	<0.001	0.0%
One year PFS	Overall	3	1.03 (1.00, 1.06)	0.035	0.0%
Two years PFS	Overall	3	1.21 (1.04, 1.40)	0.012	62.0%
Three years PFS	Overall	4	1.26 (1.17, 1.34)	<0.001	0.0%
Five years PFS	Overall	2	1.39 (1.25, 1.55)	<0.001	0.0%
CRR	Overall	4	1.28 (1.08, 1.50)	0.003	63.4%
ORR	Overall	4	1.09 (0.88, 1.34) P	0.436	96.2%
Recurrence rate	Overall	2	0.59 (0.50, 0.71)	<0.001	0.0%
Anemia	Overall	3	1.14 (0.54, 2.38)	0.730	91.5%
Leukocytopenia	Overall	3	1.19 (0.95, 1.49)	0.140	87.30%
Radiation enteritis	Overall	3	0.96 (0.64, 1.44)	0.834	54.0%
Radiocystitis	Overall	3	0.95 (0.66, 1.36)	0.765	0.0%
Thrombopenia	Overall	2	1.06 (0.98, 1.16)	0.165	31.3%

Notes: TP = cisplatin and paclitaxel, NACT = neoadjuvant chemotherapy, CCRT = concurrent chemoradiotherapy, LACC = locally advanced cervical cancer, OS = overall survival, PFS = progression-free survival, ORR = objective response rate, CRR = complete response rate, RR = relative risk, CI = confidence interval.

The comparison of 2-year OS between LACC patients treated with TP-based NACT combined with CCRT and CCRT was evaluated by three studies. The results showed that patients treated with TP-based NACT combined with CCRT had a higher 2-year OS compared with patients treated with CCRT (RR: 1.11, 95% CI: 1.07, 1.16, *P* < 0.001) (Fig. 2b, Table 2).

Four studies evaluated the 3-year OS outcomes between patients with LACC who were treated with TP-based NACT combined with CCRT and those who received CCRT alone. The results showed that NACT with the TP regimen in combination with CCRT was associated with improved 3-year OS compared with CCRT alone (RR: 1.30, 95% CI: 1.23, 1.37, *P* < 0.001) (Fig. 2c, Table 2).

As for the comparison of 5-year OS between LACC patients treated with TP-based NACT combined with CCRT and CCRT, two studies were evaluated. A higher 5-year OS was observed in LACC patients treated with TP-based NACT combined with CCRT (RR: 1.20, 95% CI: 1.10, 1.32, *P* < 0.001) (Fig. 2d, Table 2).

#### The comparison of PFS between LACC patients treated with TP-based NACT combined with CCRT and CCRT

**Fig. 3:**
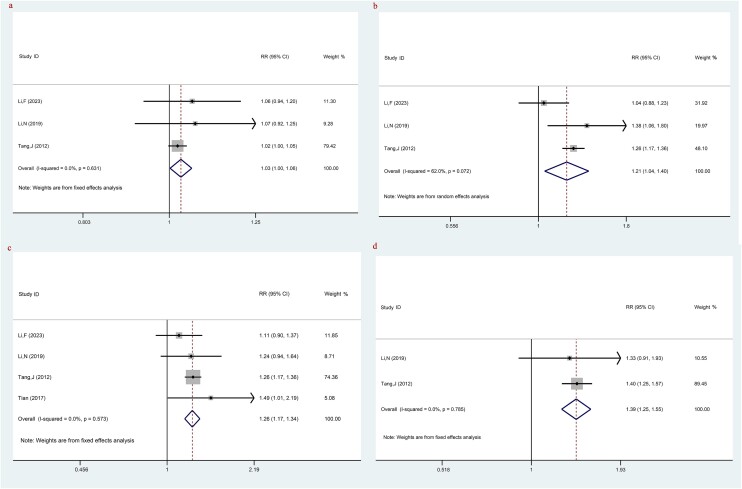
The comparison of PFS between LACC patients treating with TP-based NACT combined with CCRT and CCRT; (**a**) 1-year PFS; (**b**) 2-year PFS; (**c**) 3-year PFS; (**d**) 5-year PFS. Each row represents an individual study, with labels indicating the study name and year. The bottom row labeled ‘Overall’ represents the combined results of all studies. The horizontal lines for each study represent the 95% CI, and the square symbols indicate the RR. The size of the square is proportional to the study’s weight, with larger squares indicating greater weight. The I^2^ statistic is included to show the percentage of variability across studies due to heterogeneity. The dashed line is placed at the position of 1, representing the null hypothesis of no effect (i.e. no difference between the treatment and control groups). If a study’s CI crosses this line, it suggests that the study’s result is not statistically significant.

Three studies investigated 1-year PFS between LACC patients treated with TP-based NACT combined with CCRT and CCRT. A higher 1-year PFS was observed in LACC patients treated with TP-based NACT combined with CCRT (RR: 1.03, 95% CI: 1.00, 1.06, *P =* 0.035) (Fig. 3a, Table 2).

The comparison of 2-year PFS between LACC patients treated with TP-based NACT combined with CCRT and CCRT was investigated in three studies. The combination therapy of TP-based NACT with CCRT may be more effective in prolonging 2-year PFS for LACC patients compared with CCRT alone, based on the 2-year PFS data (RR: 1.21, 95% CI: 1.04, 1.40, *P* = 0.012) (Fig. 3b, Table 2).

Four studies examined 3-year PFS by comparing between LACC patients treated with TP-based NACT combined with CCRT and CCRT. The results indicated that the combination of TP-based NACT with CCRT may be associated with a better 3-year PFS outcome compared with CCRT alone (RR: 1.26, 95% CI: 1.17, 1.34, *P* < 0.001) (Fig. 3c, Table 2).

For the 5-year PFS, two studies were included. The result implied that the treatment regime of TP-based NACT combined with CCRT was associated with a better PFS over a 5-year period compared with the CCRT alone (RR: 1.39, 95% CI: 1.25, 1.55, *P* < 0.001) (Fig. 3d, Table 2).

#### The comparison of CRR between LACC patients treated with TP-based NACT combined with CCRT and CCRT

**Fig. 4:**
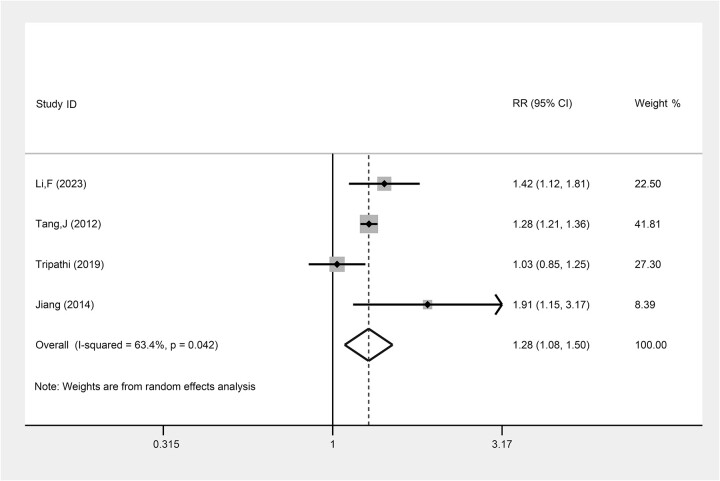
The comparison of CRR between LACC patients treating with TP-based NACT combined with CCRT and CCRT. Each row represents an individual study, with labels indicating the study name and year. The bottom row labeled ‘Overall’ represents the combined results of all studies. The horizontal lines for each study represent the 95% CI, and the square symbols indicate the RR. The size of the square is proportional to the study’s weight, with larger squares indicating greater weight. The I^2^ statistic is included to show the percentage of variability across studies due to heterogeneity. The dashed line is placed at the position of 1, representing the null hypothesis of no effect (i.e. no difference between the treatment and control groups). If a study’s CI crosses this line, it suggests that the study’s result is not statistically significant.

There were four individual studies that specifically focused on evaluating the CRR in LACC patients treated with TP-based NACT combined with CCRT and those treated with CCRT alone. The result indicated that TP-based NACT combined with CCRT was statistically significantly associated with a higher likelihood of CRR compared with CCRT alone (RR: 1.28, 95% CI: 1.08, 1.50, *P* = 0.003, I^2^ = 63.4%) (Fig. 4, Table 2).

#### The comparison of ORR between LACC patients treated with TP-based NACT combined with CCRT and CCRT

**Fig. 5:**
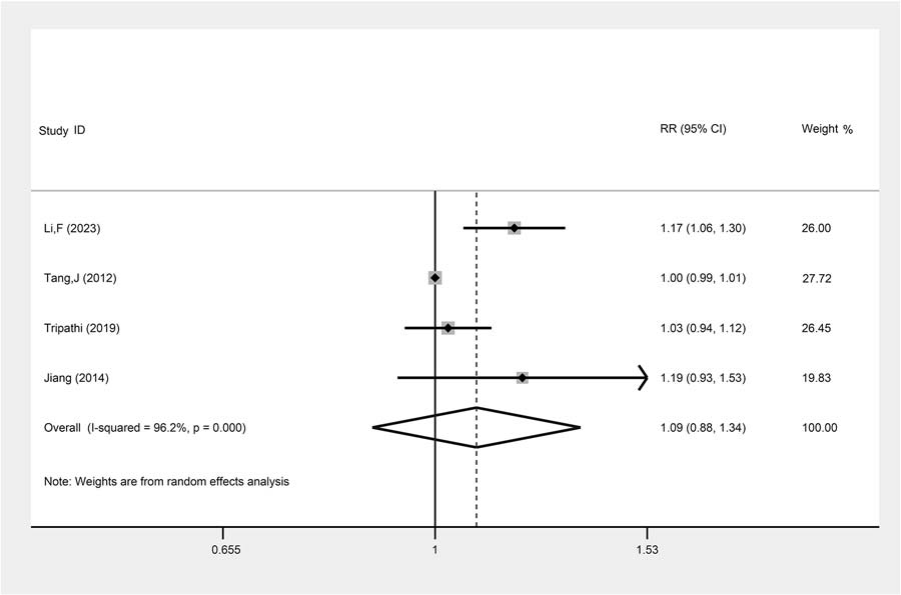
The comparison of ORR between LACC patients treating with TP-based NACT combined with CCRT and CCRT. Each row represents an individual study, with labels indicating the study name and year. The bottom row labeled ‘Overall’ represents the combined results of all studies. The horizontal lines for each study represent the 95% CI, and the square symbols indicate the RR. The size of the square is proportional to the study’s weight, with larger squares indicating greater weight. The I^2^ statistic is included to show the percentage of variability across studies due to heterogeneity. The dashed line is placed at the position of 1, representing the null hypothesis of no effect (i.e. no difference between the treatment and control groups). If a study’s CI crosses this line, it suggests that the study’s result is not statistically significant.

The comparison of the ORR between LACC patients treated with TP-based NACT combined with CCRT and those treated with CCRT alone was assessed in four studies. The result indicated that while there was a trend toward a higher ORR with the TP-based NACT combined with CCRT, the evidence was not statistically significant (RR: 1.09, 95% CI: 0.88, 1.34, *P* = 0.436, I^2^ = 96.2%) (Fig. 5, Table 2).

#### The comparison of recurrence rate between LACC patients treated with TP-based NACT combined with CCRT and CCRT

**Fig. 6:**
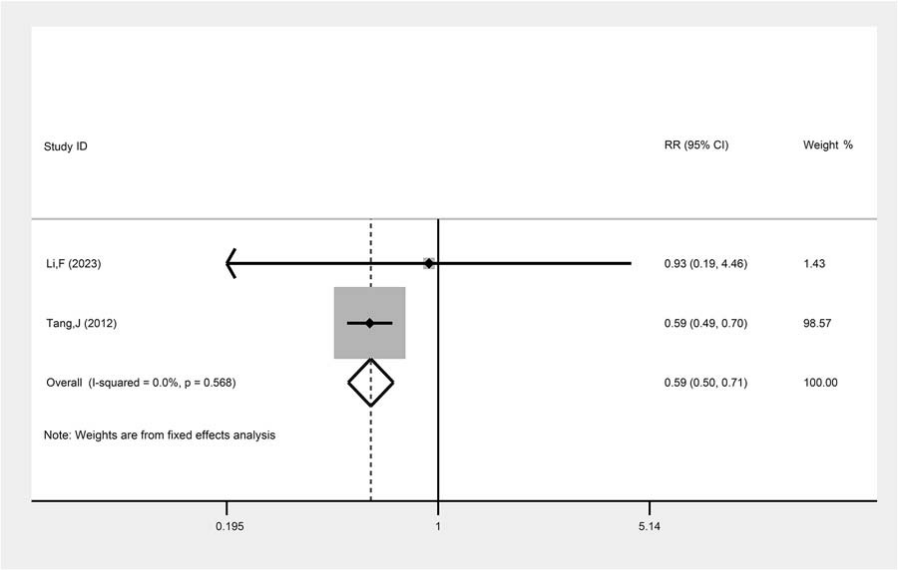
The comparison of recurrence rate between LACC patients treating with TP-based NACT combined with CCRT and CCRT. Each row represents an individual study, with labels indicating the study name and year. The bottom row labeled ‘Overall’ represents the combined results of all studies. The horizontal lines for each study represent the 95% CI, and the square symbols indicate the RR. The size of the square is proportional to the study’s weight, with larger squares indicating greater weight. The I^2^ statistic is included to show the percentage of variability across studies due to heterogeneity. The dashed line is placed at the position of 1, representing the null hypothesis of no effect (i.e. no difference between the treatment and control groups). If a study’s CI crosses this line, it suggests that the study’s result is not statistically significant.

Two studies assessed the comparison of recurrence rate between LACC patients treated with TP-based NACT combined with CCRT and CCRT alone. The result suggested that TP-based NACT followed by CCRT was more effective in reducing the recurrence rate in patients with LACC compared with CCRT alone (RR: 0.59, 95% CI: 0.50, 0.71, *P* < 0.001) (Fig. 6, Table 2).

#### The comparison of adverse events between LACC patients treated with TP-based NACT combined with CCRT and CCRT

**Fig. 7:**
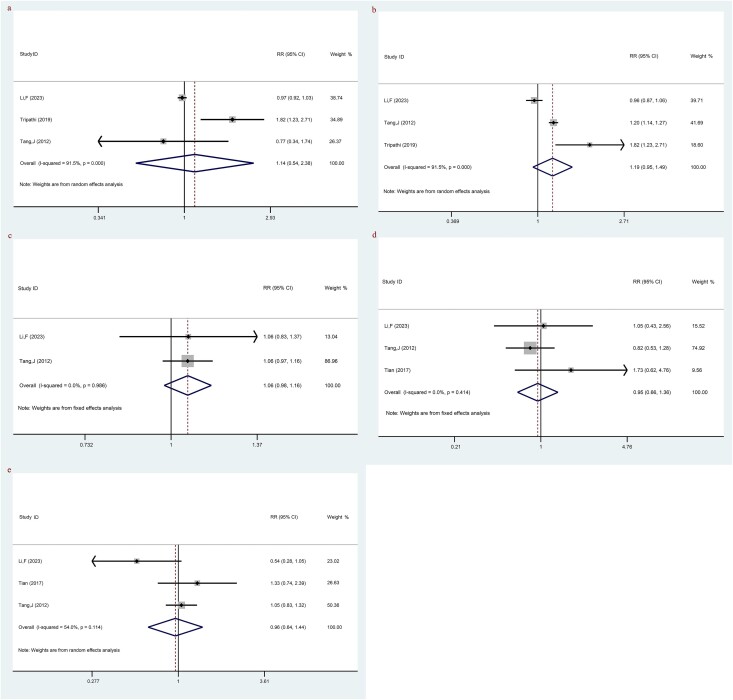
The comparison of adverse events between LACC patients treating with TP-based NACT combined with CCRT and CCRT; (**a**) anemia; (**b**) leukocytopenia; (**c**) thrombopenia; (**d**) radiocystitis; (**e**) radiation enteritis. Each row represents an individual study, with labels indicating the study name and year. The bottom row labeled ‘Overall’ represents the combined results of all studies. The horizontal lines for each study represent the 95% CI, and the square symbols indicate the RR. The size of the square is proportional to the study’s weight, with larger squares indicating greater weight. The I^2^ statistic is included to show the percentage of variability across studies due to heterogeneity. The dashed line is placed at the position of 1, representing the null hypothesis of no effect (i.e. no difference between the treatment and control groups). If a study’s CI crosses this line, it suggests that the study’s result is not statistically significant.

There were three individual studies that specifically evaluated anemia among LACC patients treated with TP-based NACT combined with CCRT and CCRT alone. Based on the available data, combining TP-NACT with CCRT did not appear to reduce the risk of anemia in patients with LACC compared with CCRT alone (RR: 1.14, 95% CI: 0.54, 2.38, *P* = 0.730) (Fig. 7a, Table 2).

Three studies assessed leukocytopenia with NACT content (TP) and CCRT alone. The result indicated that there was a trend toward a higher risk of leukocytopenia with the TP-based NACT combined with CCRT, but the evidence was not statistically significant (RR: 1.19, 95% CI: 0.95, 1.49, *P* = 0.140) (Fig. 7b, Table 2).

The thrombopenia between LACC patients treated with TP-based NACT combined with CCRT and CCRT was evaluated in two studies. There was no significant difference in thrombopenia between LACC patients treated with TP-based NACT combined with CCRT and CCRT (RR: 1.06, 95% CI: 0.98, 1.16, *P* = 0.165) (Fig. 7c, Table 2).

Three studies assessed radiocystitis between LACC patients treated with TP-based NACT combined with CCRT and CCRT. The addition of TP-based NACT to CCRT did not appear to substantially affect the risk of radiocystitis in LACC patients treated with TP-based NACT combined with CCRT compared with CCRT treatment (RR: 0.95, 95% CI: 0.66, 1.36, *P* = 0.765) (Fig. 7d, Table 2).

Radiation enteritis between LACC patients treated with TP-based NACT combined with CCRT and CCRT was assessed in three studies. The result indicated that there was no significant difference between TP-based NACT combined with CCRT and CCRT in radiation enteritis (RR: 0.96, 95% CI: 0.64, 1.44, *P* = 0.834) (Fig. 7e, Table 2).

### A systematic review of the clinical efficacy and safety of cisplatin and gemcitabine-based NACT combined with CCRT in patients with LACC

In the study conducted by da Costa *et al.* [9], the incorporation of NACT, which consists of cisplatin and gemcitabine, into the standard CCRT did not show superiority and might even be inferior to CCRT alone for the treatment of LACC.

### A systematic review of the clinical efficacy and safety of cisplatin and irinotecan based NACT combined with CCRT in patients with LACC

In the study by Zhong *et al.* [17], the impact of CCRT followed by cisplatin and irinotecan on cervical cancer patients was found to be superior to CCRT alone, and it was associated with a reduced incidence of adverse reactions.

## DISCUSSION

In this analysis, which incorporated data from eight relevant studies, we evaluated the therapeutic effectiveness of NACT followed by CCRT in individuals with LACC. Our findings indicate that LACC patients who underwent treatment with TP-based NACT in conjunction with CCRT demonstrated improved OS at 2, 3 and 5 years, as well as PFS at 1, 2, 3 and 5 years when compared with patients who received CCRT alone. Moreover, the TP-based NACT in conjunction with the CCRT group achieved a higher CRR and exhibited a lower rate of disease recurrence. Notably, we observed no significant differences in the risk of adverse effects including anemia, leukopenia, thrombocytopenia, radiocystitis and radiation enteritis between the group treated with TP-based NACT combined with CCRT and the group treated with CCRT alone.

The result showed that LACC patients treated with TP-based NACT combined with CCRT had better 2-, 3- and 5-year OS, 1-, 2-, 3- and 5-year PFS, higher likelihood of CRR, and reduced recurrence rate compared with patients treating with CCRT. Several previous studies supported our findings. A clinical trial has determined that the combination of NACT with extensive CCRT can lead to better outcomes for individuals with stage IB and II cervical cancer who exhibit positive para-aortic lymph nodes [21]. A retrospective analysis of a group of 713 individuals with locally advanced squamous cell carcinoma of the cervix revealed that patients who received CCRT after NACT experienced a significantly enhanced disease-free survival compared with those who underwent CCRT alone [22]. A retrospective analysis of 207 stage IIB-IIIB cervical cancer patients who underwent 2–4 cycles of platinum-based NACT before CCRT showed that the 5-year survival rates were 84% for stages IIB-IIIA and 61% for stage IIIB, both of which were higher than those achieved with CCRT alone [23]. In addition to the mentioned studies, there is also research that has investigated the efficacy of NACT combined with CCRT, specifically using a regimen that includes TP. The findings from a clinical trial indicate that administering four cycles of NACT with weekly cisplatin at a dosage of 40 mg/m2 in conjunction with paclitaxel at 60 mg/m2, followed by CCRT, is a viable treatment approach and has demonstrated a favorable response rate [5]. A pilot study has found that the use of dose-dense weekly TP as NACT, followed by standard CCRT, was associated with a high response rate in patients with LACC [24]. The use of NACT prior to CCRT may serve to eradicate subclinical distant metastases, reduce tumor size and correct distortions in pelvic anatomy, thereby potentially leading to improved subsequent treatment outcomes [5].

The observation from a regional oncology center’s research underscores that the categorization of NACT significantly correlates with the survival rates of patients diagnosed with cervical cancer who are subjected to radiotherapeutic interventions [22]. It is implied that the selection of distinct chemotherapeutic medications or the application of varied treatment protocols may potentially yield differential effects on the survival prognosis of these patients. A phase II study has demonstrated that dose-dense NACT with weekly paclitaxel and carboplatin, subsequently followed by CCRT could achieve a favorable response rate in LACC [25]. Due to the limitations of the included literature, we were unable to conduct a further analysis on the efficacy of different NACT regimens combined with CCRT for patients with LACC. However, we advocate for future research to potentially delve into the comparative effectiveness of various NACT combinations, including but not limited to paclitaxel and carboplatin, when administered in conjunction with CCRT for the LACC patient population.

We found no notable disparities in the risk of adverse effects such as anemia, leukopenia, thrombocytopenia, radiocystitis and radiation enteritis when comparing the group that received TP-based NACT followed by CCRT to the group that underwent CCRT alone. While NACT can have potential benefits, such as those mentioned previously, it is not without potential negative impacts. A systematic review has identified that NACT in the treatment of LACC is associated with mild early toxicities [26]. Leukopenia and neutropenia are the most common side effects, while late toxicities are generally also mild and are predominantly related to bladder dysfunction and vaginal stenosis. One study found that the most common early adverse events of NACT+ CCRT were bone marrow suppression, gastrointestinal symptoms and fatigue [5]. The findings highlight the importance of considering both the potential benefits and the possible negative impacts when deciding on a treatment plan. While NACT may provide certain advantages, it is essential to monitor and manage the side effects to ensure patient well-being.

The randomized study INTERLACE (a phase III multicenter trial of weekly induction chemotherapy (IC) followed by standard chemoradiation versus standard chemoradiation alone in patients with LACC) of NACT presented recently, has shown significant improvement in survival with the use of six cycles of weekly carboplatin and paclitaxel. While both our meta-analysis and the INTERLACE study demonstrate the benefits of integrating chemotherapy with CCRT for LACC patients, the comprehensive nature of our meta-analysis offers a broader and more generalized validation of the TP regimen combined with CCRT, highlighting its overall efficacy and safety across various studies and patient groups. This generalizability is crucial for guiding clinical practice and policy-making, as it suggests that the combined treatment can be beneficial in diverse clinical settings. On the other hand, the INTERLACE study’s controlled trial design provides specific, high-quality evidence supporting the IC approach, which is essential for establishing new treatment protocols.

The clinical significance of a meta-analysis on the efficacy of NACT combined with CCRT for the treatment of LACC can be outlined as follows: first, the meta-analysis provides a comprehensive assessment of the effectiveness of the combination of NACT and CCRT in treating LACC, aiding clinicians in choosing the most appropriate treatment plan for patients. Second, the aggregated analysis allows for a better understanding of the treatment’s safety profile, including the types and incidence of treatment-related adverse events and the risk of serious adverse events. Third, the systematic evaluation of results from various studies can facilitate consensus on treatment strategies for LACC, both internationally and regionally, and contribute to the updating and improvement of treatment guidelines. Fourth, the meta-analysis can reveal existing challenges and issues in current treatments, providing direction for future research, such as exploring new biomarkers, optimizing treatment regimens or developing novel therapeutic approaches. Fifth, this study may provide evidence-based support for health decision-makers to make more informed health policy and resource allocation decisions within the constraints of limited resources.

The limitations of this study should be mentioned. First, the study is limited by the geographical scope of the included trials, with the majority of the countries represented being China and India. This geographic bias may affect the generalizability of the findings. Second, in acknowledging the established efficacy of CCRT as the standard of care for LACC, our study also takes into account the stark reality of healthcare disparities that affect many regions around the world. One such disparity is the limited access to radiotherapy facilities, which can lead to significant delays in the initiation of CCRT for patients in need. This limitation, in turn, has prompted the consideration of NACT as an alternative treatment option for those who would otherwise face prolonged waiting periods. While NACT may provide temporary relief or some therapeutic benefit, it is not equivalent to the definitive treatment that CCRT offers. We acknowledge that the choice between NACT and CCRT is often dictated by factors beyond clinical judgment, such as resource availability and patient accessibility. Third, a significant limitation of our study is the overall suboptimal quality of the RCTs included in the meta-analysis. The majority of these RCTs exhibited a higher risk of bias, which could have influenced the outcomes and potentially limited the robustness of our findings. The presence of methodological weaknesses, such as selection and reporting biases, may have introduced confounding factors that affect the reliability of the conclusions drawn. This limitation suggests that our findings should be interpreted with caution, and highlights the need for higher quality, well-conducted RCTs to provide more definitive evidence on the efficacy and safety of TP-based NACT combined with CCRT in LACC. Fourth, Subgroup analyses were not possible for certain variables, such as the dosage and frequency of NACT, and the classification of cervical cancer, due to insufficient data in the literature. This limitation restricts the ability to draw more granular conclusions about the effects of specific treatment regimens and patient subgroups. Fifth, we recognize that the emergence of the INTERLACE trial results may influence the generalizability of our findings and the interpretation of our conclusions. While our study provides valuable insights based on the current standard of care, the introduction of the TC regimen as a promising alternative necessitates further investigation into its implications for patient outcomes and treatment protocols. Sixth, the number of studies included in the analysis is small, which may affect the statistical power of the findings. The limitations outlined above should be taken into consideration when interpreting the results of the meta-analysis.

## CONCLUSION

Our study results support the use of NACT with a TP regimen, followed by CCRT, as a potentially effective treatment strategy for improving the prognosis of patients with LACC. The findings of our study encourage further research into the optimal integration of NACT and CCRT. This research may contribute to the development of treatment protocols that utilize NACT combined with CCRT, with the aim of reducing the burden of LACC.

## Supplementary Material

Supplementary_Figure_1_rrae073

Supplementary_Figure_2_rrae073

Supplementary_file_1_rrae073

Supplementary_file_2_rrae073

## Data Availability

The datasets used and/or analysed during the current study available from the corresponding author on reasonable request.
