# Supplementary figures and images for: Clinical efficacy and safety of neoadjuvant chemotherapy with paclitaxel and cisplatin in combination with concurrent chemoradiotherapy for locally advanced cervical cancer: a systematic review and meta-analysis
Source: J Radiat Res. 2024 Oct 5;65(6):733–43. doi: 10.1093/jrr/rrae073 (PMC11630013; doi:10.1093/jrr/rrae073)

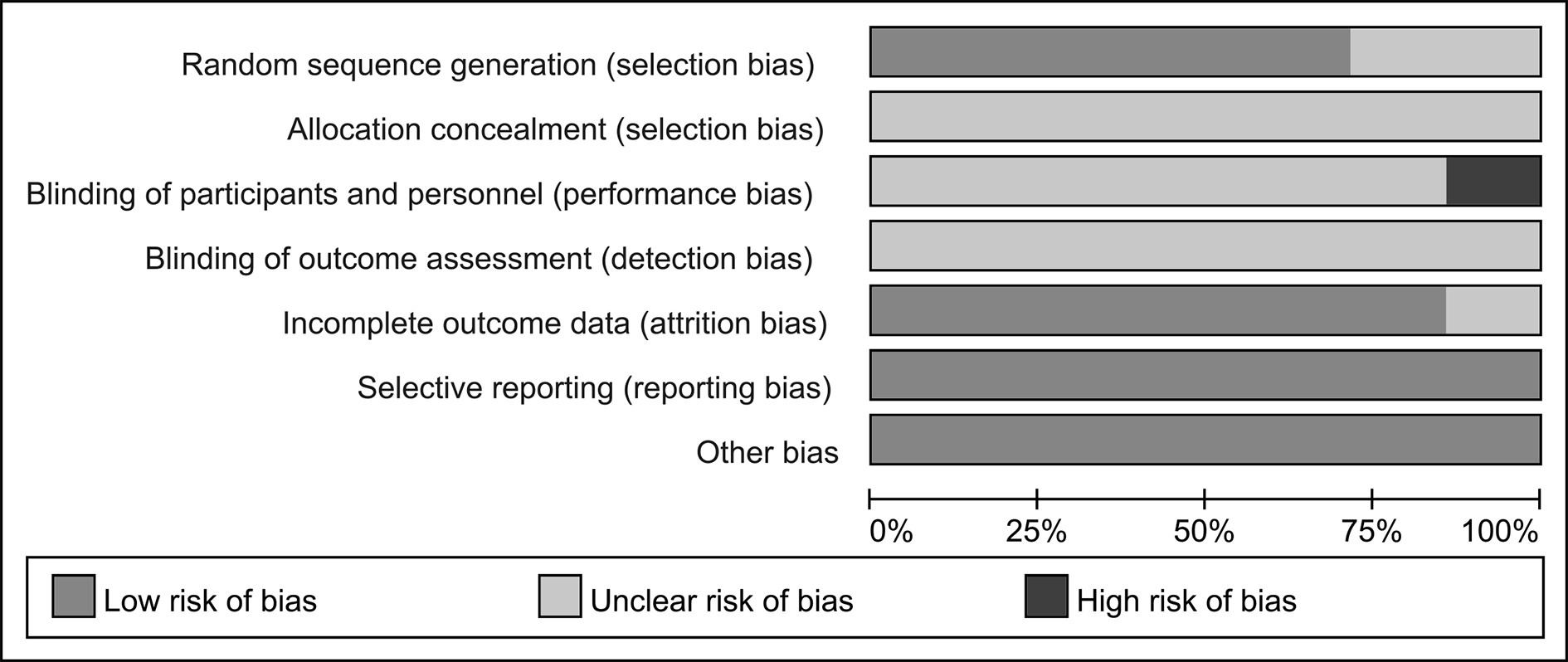

Supplement: Supplementary_Figure_1_rrae073 [file supplementary_figure_1_rrae073.jpeg]

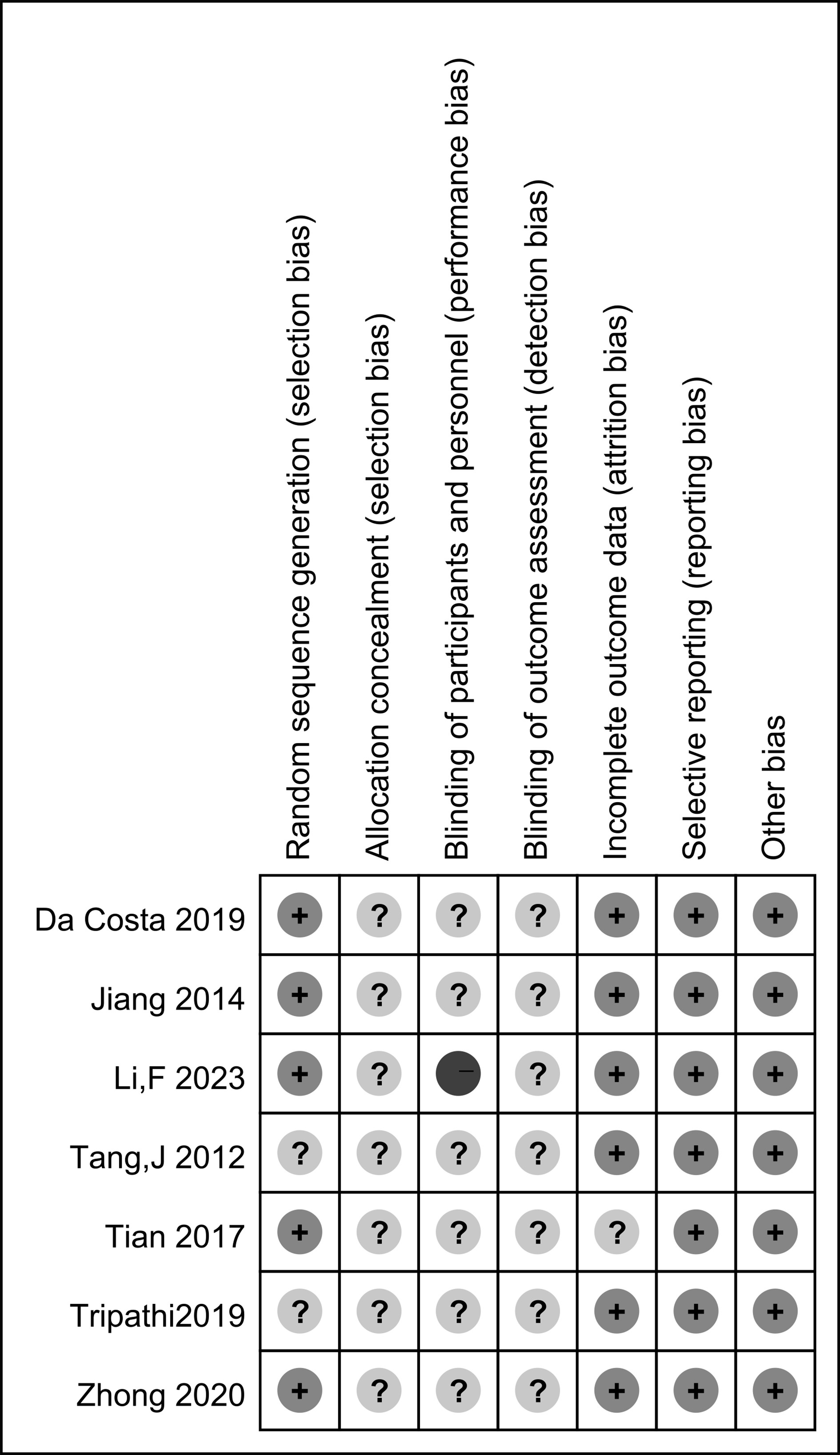

Supplement: Supplementary_Figure_2_rrae073 [file supplementary_figure_2_rrae073.jpeg]
