# Supplementary material for: Clinical efficacy and safety of neoadjuvant chemotherapy with paclitaxel and cisplatin in combination with concurrent chemoradiotherapy for locally advanced cervical cancer: a systematic review and meta-analysis
Source: J Radiat Res. 2024 Oct 5;65(6):733–43. doi: 10.1093/jrr/rrae073 (PMC11630013; doi:10.1093/jrr/rrae073)
Supplement: Supplementary_file_1_rrae073 [file supplementary_file_1_rrae073.docx]

The PubMed search strategy, designed to identify relevant studies for our research, was constructed as follows: “cervical cancer” OR “Cervical Neoplasm, Uterine” OR “Neoplasm, Uterine Cervical” OR “Uterine Cervical Neoplasm” OR “Neoplasms, Cervical” OR “Cervical Neoplasm*” OR “Neoplasms, Cervix” OR “Cervix Neoplasm*” OR “Neoplasm, Cervix” OR “Cancer of the Uterine Cervix” OR “Cancer of the Cervix” OR “Cervical Cancer*” OR “Cancer, Cervical” OR “Uterine Cervical Cancer*” OR “Cancer, Uterine Cervical” OR “Cervical Cancer, Uterine” OR “Cancer of Cervix” OR “Cervix Cancer” OR “Cervix Cancer” AND “Chemoradiation” OR “Chemoradiotherapies” OR “Radiochemotherapy*” OR “Concurrent Chemoradiotherapy*” OR “Chemoradiotherapy*, Concurrent” OR “Synchronous Chemoradiotherapy*” OR “Chemoradiotherapy*, Synchronous” OR “Radiochemotherapy*, Concurrent” OR “Concurrent Radiochemotherapy*” OR “Concomitant Chemoradiotherapy*” OR “Chemoradiotherapy*, Concomitant” OR “Concomitant Radiochemotherapy*” OR “Radiochemotherapy*, Concomitant” OR “concomitant chemoradiation” OR “radiotherapy*” OR “Radiation Therapy*” OR “Therapy*, Radiation” OR “Radiation Treatment*” OR “Treatment, Radiation” OR “Radiotherapy*, Targeted” OR “Targeted Radiotherapy*” OR “Targeted Radiation Therapy*” OR “Therapy*, Targeted Radiation” OR “Radiation Therapy*, Targeted” OR “concurrent chemoradiation” OR “CCRT” OR “CRT” OR “chemotherapy” OR “Drug Therapy, Adjuvant” OR “Adjuvant Chemotherapy” OR “Adjuvant Drug Therapy” OR “adjuvant chemotherapy” AND “cis Platinum” OR “cis Platinum” OR “Platino” OR “Carboplatin” OR “Paclitaxel” OR “Taxol” OR “Topotecan” OR “Neoadjuvant Chemotherapy” OR “NACT” OR “Neoadjuvant Therapy*” OR “Therapy, Neoadjuvant” OR “Neoadjuvant Treatment*” OR “Treatment, Neoadjuvant” OR “Neoadjuvant Chemoradiotherapy*” OR “Chemoradiotherapy, Neoadjuvant” OR “Neoadjuvant Chemoradiation Therapy*” OR “Chemoradiation Therapy, Neoadjuvant” OR “Therapy, Neoadjuvant Chemoradiation” OR “Neoadjuvant Chemoradiation Treatment*” OR “Chemoradiation Treatment, Neoadjuvant” OR “Treatment, Neoadjuvant Chemoradiation” OR “Neoadjuvant Chemoradiation*” OR “Chemoradiation, Neoadjuvant” OR “Neoadjuvant Radiotherapy*” OR “Radiotherapy, Neoadjuvant” OR “Neoadjuvant Radiation Treatment*” OR “Radiation Treatment, Neoadjuvant” OR “Treatment, Neoadjuvant Radiation” OR “Neoadjuvant Radiation Therapy*” OR “Radiation Therapy, Neoadjuvant” OR “Therapy, Neoadjuvant Radiation” OR “Neoadjuvant Radiation*” OR “Radiation, Neoadjuvant” OR “Neoadjuvant Chemotherapy*” OR “Chemotherapy, Neoadjuvant” OR “Neoadjuvant Chemotherapy Treatment*” OR “Chemotherapy Treatment, Neoadjuvant” OR “Treatment, Neoadjuvant Chemotherapy” OR “Neoadjuvant Systemic Therapy*” OR “Systemic Therapy, Neoadjuvant” OR “Therapy, Neoadjuvant Systemic” OR “Neoadjuvant Systemic Treatment*” OR “Systemic Treatment, Neoadjuvant” OR “Treatment, Neoadjuvant Systemic”
