# Supplementary material for: Clinical efficacy and safety of neoadjuvant chemotherapy with paclitaxel and cisplatin in combination with concurrent chemoradiotherapy for locally advanced cervical cancer: a systematic review and meta-analysis
Source: J Radiat Res. 2024 Oct 5;65(6):733–43. doi: 10.1093/jrr/rrae073 (PMC11630013; doi:10.1093/jrr/rrae073)
Supplement: Supplementary_file_2_rrae073 [file supplementary_file_2_rrae073.docx]

Supplementary file 2 Quality of the included studies assessed by NOS

| Author | Year | Selection of study population | Comparability between groups | Outcome measurement | QA |
| --- | --- | --- | --- | --- | --- |
| Li, N | 2019 | 4 | 1 | 2 | 7 |

Notes: NOS: Newcastle-Ottawa Scale; QA: quality assessment.
